# *In situ* flexible wearable tomato growth sensor: monitoring of leaf physiological characteristics

**DOI:** 10.3389/fpls.2025.1546373

**Published:** 2025-03-21

**Authors:** Longjie Li, Junxian Guo, Shuai Wang, Wei Zhou, Yanjun Huo, Gongyong Wei, Yong Shi, Lingyu Li

**Affiliations:** ^1^ College of Mechanical and Electrical Engineering, Xinjiang Agricultural University, Urumqi, China; ^2^ Key Laboratory of Xinjiang Intelligent Agricultural Equipment, Xinjiang Agricultural University, Urumqi, China

**Keywords:** crop phenotyping and estimation, chlorophyll, water content, spectrum, precision farming

## Abstract

*In situ* real-time monitoring of physiological information during crop growth (such as leaf chlorophyll values and water content) is crucial for enhancing agricultural production efficiency and crop management practices. In traditional agricultural monitoring, commonly used measurement methods, such as chemical analysis for determining leaf chlorophyll values and drying methods for measuring water content, are all non-*in situ* measurement techniques. These methods not only risk damaging the plants but may also impact plant growth and health. Furthermore, the complex setup of traditional spectrometers complicates the data collection process, which limits their practical application in plant monitoring. Therefore, there is an urgent need to develop a novel, user friendly, and plant-safe monitoring technology to improve agricultural management efficiency. To this end, this study proposes a novel wearable flexible sensor designed for *in situ* real-time monitoring of leaf chlorophyll values and water content. This sensor is lightweight, portable, and allows for flexible placement, enabling continuous monitoring by conforming to plant surfaces. Its spectral response covers multiple bands from near ultraviolet to near infrared, and it is equipped with an active light source ranging from ultraviolet to infrared to enable efficient measurements under various environmental conditions. In addition, the sensor is securely attached to the underside of the leaf using a magnetic suction method, ensuring long-term stable *in situ* monitoring, thus continuously collecting important physiological information throughout the crop growth cycle. Analysis of the sensor-collected data reveals that for leaf chlorophyll, Gaussian process regression shows the best prediction performance during multi-spectral scattering correction, with R_c_^2^ of 0.8261 and RMSEc of 1.7444 on the training set; the performance on the test set is Rp² of 0.7155 and RMSE_p_ of 2.0374. Meanwhile, for leaf water content, across various data preprocessing scenarios, gradient boosting regression can effectively predict it, yielding Rc² of 0.9401 and RMSEc of 0.0028 on the training set; the performance on the test set is R_c_^2^ of 0.6667 and RMSE_p_ of 0.0067.

## 1 Introduction

With the rapid growth of the global population and the severe challenges posed by climate change, agriculture is facing unprecedented pressure (Sivakumar, 2021). In this context, enhancing crop production efficiency, ensuring food security, and achieving sustainable development have become urgent tasks worldwide (Gil et al., 2019). According to the Food and Agriculture Organization (FAO), global food demand is projected to increase by more than 70% by 2050 compared to 2010 (Dawson et al., 2016). To address this trend, precision agriculture has increasingly become a key strategy for achieving sustainable agricultural production (Trivelli et al., 2019). In precision agriculture, the physiological information of crops (such as photosynthesis, transpiration, and nutrient uptake) forms the basis for optimizing management decisions. These physiological traits directly impact crop growth and yield, making real-time monitoring of the physiological status of crops particularly important. The development of modern sensor technology provides new solutions for such monitoring, especially with the emergence of wearable flexible sensors, which makes it possible to track their physiological traits *in situ* within the natural environment of crop growth.

In agricultural production, crop growth status is closely associated with physiological characteristics, particularly leaf-derived physiological information such as SPAD values (Soil Plant Analysis Development index, indicating relative chlorophyll content) and water content, which are crucial for improving crop yield and quality (Latifinia and Eisvand, 2022). Effectively monitoring these physiological features and implementing appropriate management practices can not only support crop photosynthesis and transpiration but also optimize overall growth and development (Kang et al., 2021). However, traditional measurement methods, such as extracting leaf chlorophyll through chemical methods using organic solvents like ethanol, methanol, and acetone (Ngcobo et al., 2024), or calculating the water content by drying fresh plant leaves in an oven (Zhou et al., 2021), present numerous limitations. These methods are complex in operation process, time-consuming, and can easily damage plants, which may interfere with the normal growth of crops and directly affect the efficiency and effectiveness of agricultural management (Muñoz-Huerta et al., 2013).

To address the limitations of traditional measurement methods, researchers are continuously exploring more efficient, portable, and non-destructive monitoring approaches. Spectral analysis technology has recently gained prominence in detecting plant physiological characteristics due to its high speed, non-destructive nature, and real-time monitoring capabilities (Zahir et al., 2022). Through non-contact measurements in the near-infrared, visible, and ultraviolet spectral regions, researchers can rapidly obtain information on crop chlorophyll content, water content, and other physiological indicators, which provides timely feedback on crop health and supports informed decision-making (Tripodi et al., 2018). However, existing measurement methods using traditional spectral instruments, for example, when using a portable ground object spectrometer to invert and predict the leaf chlorophyll concentration, the collection of leaf spectral reflection data outdoors requires a computer to complete (Sun et al., 2021); when using hyperspectral remote sensing technology to predict the leaf water content, it is necessary to obtain hyperspectral image data of the growth status of plants with the help of drones (Guo et al., 2024). These methods generally have the problems of large volume and complex operation, making it difficult to apply them conveniently and quickly on site. Moreover, restricted by the operation requirements of the equipment, they often cannot meet the needs of long-term monitoring of crops (Yang et al., 2021).

In light of this situation, wearable sensors have emerged as next-generation monitoring tools with significant development potential. Their lightweight and flexible characteristics allow sensors to conform to plant surfaces, thus minimizing the impact on plant growth and ensuring stable monitoring results across varying environmental conditions (Yin et al., 2021). For example, a highly stretchable wearable sensor inspired by origami, which is pasted on the front side of the plant leaf, can monitor the microclimate information such as the temperature and humidity of the leaf *in situ* (Zhang et al., 2024); a wearable Au@PET electrode made by magnetron sputtering of gold nanoparticles on a PET film, which is attached to both sides of the plant leaf, can complete the *in situ* monitoring of the leaf humidity (Peng et al., 2024). Therefore, developing an innovative wearable spectral sensor will provide a novel solution for *in situ* monitoring of the physiological characteristics of crop leaves.

The goal of this study is to design a new type of wearable flexible spectral sensor aimed at achieving long-term *in situ* monitoring of key physiological information during crop growth, particularly chlorophyll content and leaf water content. The design of this sensor uses flexible polyimide (PI) material and copper foil circuit material, combined with an adhesive, to create a thin flexible circuit board with a thickness of only 0.11 mm. The active area of this circuit board is 1.2 cm × 1.1 cm, which can accommodate the application needs of leaf samples from plants of various sizes, ensuring flexibility in monitoring crop growth conditions. Using tomato leaves as experimental samples, this study employs random sampling methods from different positions of various plants to perform SPAD measurements, collect spectral data, and gather water content data. Combining multiple denoising preprocessing techniques and utilizing various machine learning regression algorithms, we verify the predictive capacity of the spectral data collected by the sensor for leaf SPAD and water content, further assessing the feasibility of wearable measurements.

The innovation of this study lies in the application of a wearable, flexible spectral sensor for monitoring crop physiological characteristics, effectively addressing the limitations of traditional agricultural monitoring technologies in long-term observation. This non-destructive, real-time monitoring approach provides farmers with timely feedback, facilitating more scientific and effective crop management. Moreover, lightweight design of the sensor minimizes its impact on plant growth, promoting its suitability for large-scale agricultural applications in the future.

## 2 Materials and methods

### 2.1 Overview of experimental methods

**Figure 1:**
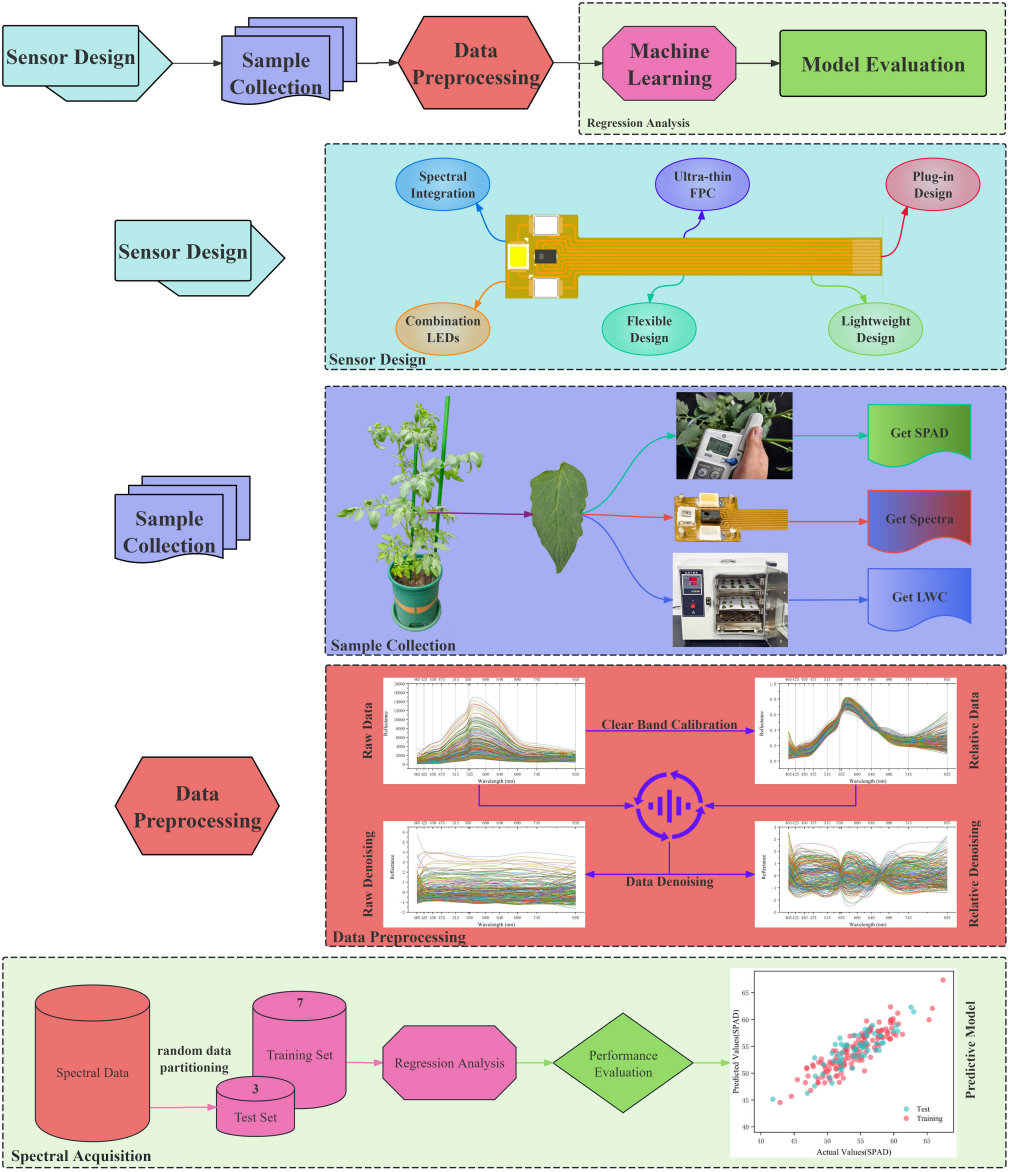
General flow of the experimental program.

This study aims to develop an integrated, flexible, wearable spectral sensor for leaves to achieve real-time monitoring of key physiological data during crop growth. As shown in **Figure 1**, the research methodology can be summarized into five main steps: sensor design, sample collection, data preprocessing, machine learning modeling, and result analysis with model prediction.

**Figure 2:**
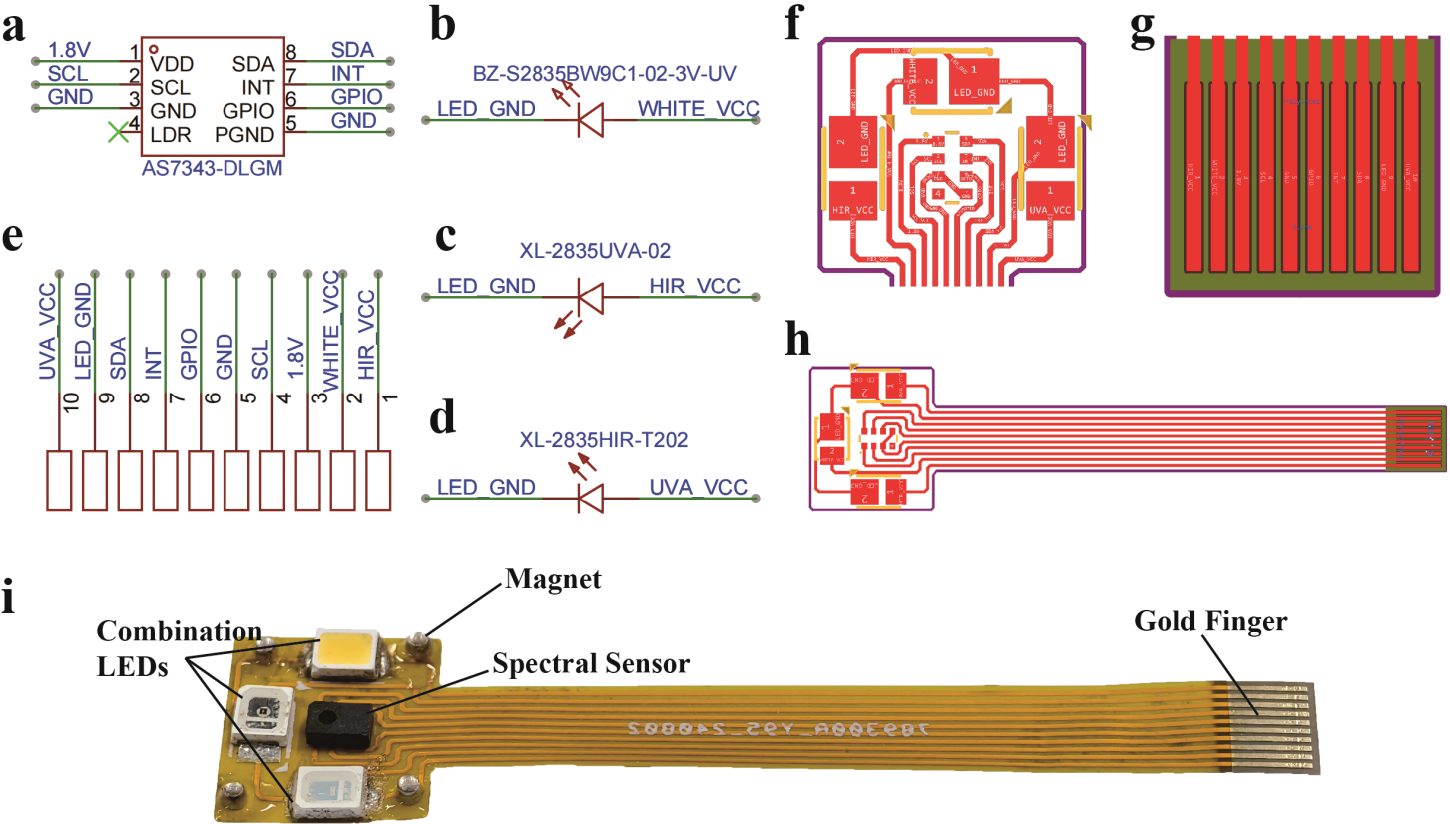
Sensor circuit design. **(a)** AS7343; **(b)** White LED; **(c)** Infrared LED; **(d)** UV LED; **(e)** Gold Finger; **(f)** FPC Pad; **(g)** FPC Gold Finger; **(h)** FPC Circuit; **(i)** Spectral Sensor.

During the sensor design phase, the circuit schematic (**Figure 2a–e**), circuit layout diagram (**Figure 2f–h**), and physical implementation (**Figure 2i**) selected lightweight and durable materials to achieve an ultra-thin, flexible FPC leaf spectral sensor design, maximizing both wearing comfort and long-term stability of the sensor. The sensor integrates multiple LED lights across various spectral ranges as active light sources, allowing it to capture the spectral characteristics of crops at different growth stages, thus providing rich spectral data for subsequent analysis.

During the sample collection phase, tomatoes (Solanum lycopersicum) were chosen as the study subject due to their high sensitivity to factors such as temperature, humidity, light, and soil conditions. This sensitivity makes them excellent candidates for sensor-based monitoring systems that can help maintain ideal growing conditions and enhance crop quality. Key physiological indicators, including photosynthetic chlorophyll content, leaf reflectance spectral data, and leaf water content, were quantitatively measured using equipment such as a SPAD meter, spectral sensor, and drying oven.

In the data preprocessing stage, the sensor’s Clear band was used to calibrate the raw spectral data, extracting the relative spectral data from the sensor. Additionally, various denoising techniques were employed to improve the signal-to-noise ratio of the spectral data, specifically including Multiplicative Scatter Correction, Standard Normal Variate, and Wavelet Denoising. These techniques effectively remove environmental noise and instrument errors, enhancing the accuracy and reliability of subsequent analyses.

During the machine learning modeling phase, the spectral data were randomly divided at a ratio of 7:3 to construct multiple regression models, including Gradient Boosting Regression (GBR), Gaussian Process Regression (GPR), and Support Vector Regression (SVR). To further improve the accuracy of physiological parameter predictions, ensemble methods such as Voting Regressor and Stacking Regressor were employed. These techniques enhance overall predictive performance by combining the predictive capabilities of different models.

Finally, in the result analysis and optimization phase, a comprehensive evaluation of the model’s performance was conducted, including metrics such as Root Mean Square Error (RMSE) and the coefficient of determination (R²), ensuring that the constructed models possess good generalization capabilities. Based on the evaluation results, the data processing workflow was continually optimized to improve the system’s adaptability and application potential, providing a scientific basis for future crop growth monitoring.

### 2.2 Design of wearable spectral sensor

**Figure 3:**
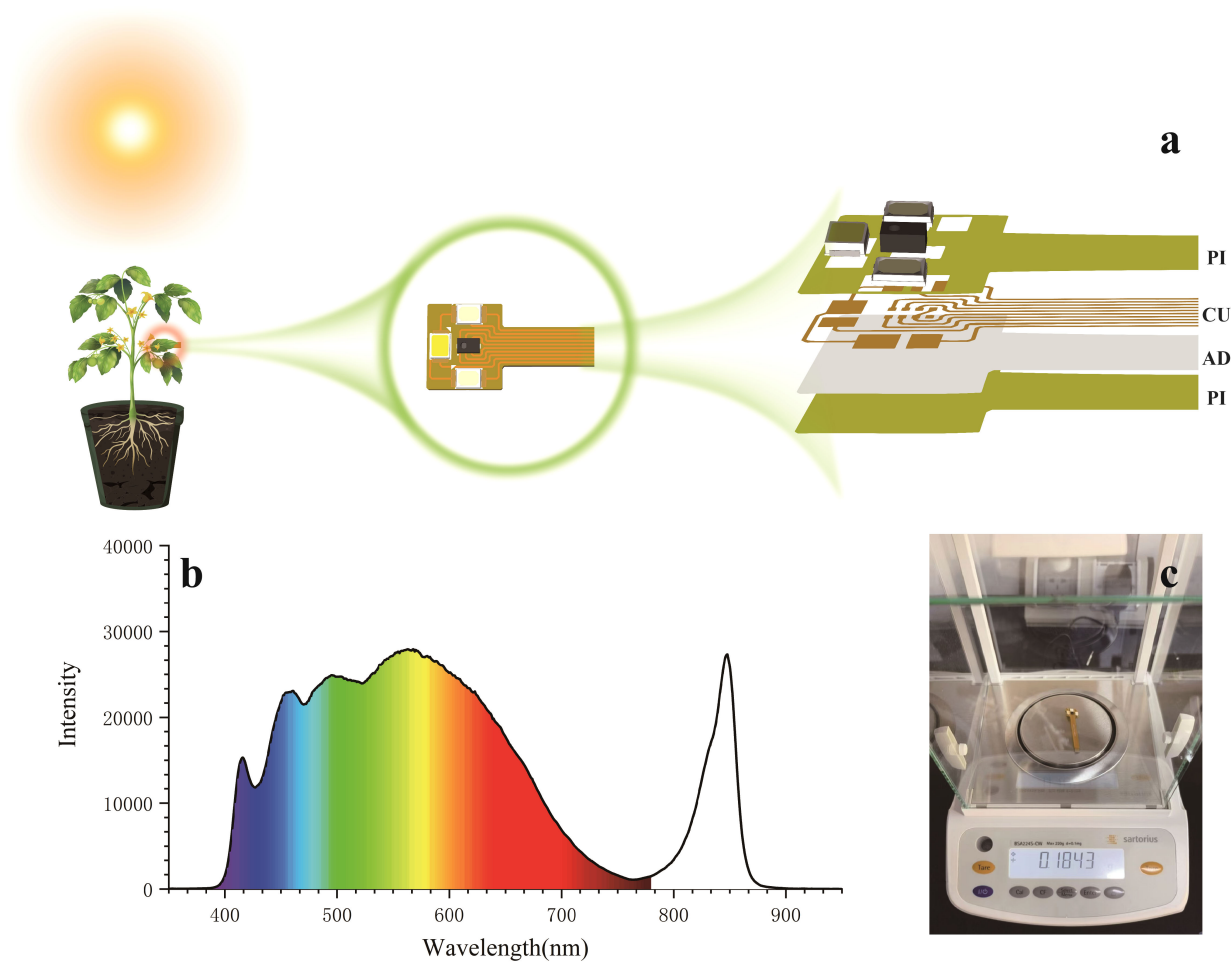
Spectral sensor design. **(a)** Core structure of the sensor; **(b)** Spectrum of the combined light sources; **(c)** Weight of the sensor.

To achieve long-term *in situ* monitoring of the physiological characteristics of crop growth, this study designed an innovative wearable flexible spectral sensor. The sensor aims to minimize its impact on crop growth while ensuring stable long-term monitoring capabilities. **Figure 3a** shows the core structure of the sensor, which consists of two layers of flexible polyimide (PI) substrate material. It is tightly bonded to copper (Cu) wires using acrylic (AD) adhesive, forming a thin single-layer flexible circuit board (FPC) with a thickness of only 0.11 mm and an active area measuring 1.2 cm × 1.1 cm. This design can adapt to plants with leaves of different sizes, meeting the crop monitoring needs throughout various growth stages.

To cover the spectral monitoring bands of the sensor, a combination of infrared, ultraviolet, and visible light LED sources was employed. As shown in **Figure 3b**, spectral data of the combined light sources were collected using a spectrometer (monitoring bandwidth 200-1200 nm). The data indicate that this combination of light sources can provide a full spectrum from near-ultraviolet to visible light and into near-infrared. This combination satisfies the monitoring requirements for the AS7343 spectral sensor within the 405-855 nm spectral width, thereby ensuring effective capture of diverse spectral information during the plant growth process.

As shown in **Figure 3c**, the overall weight of the sensor measured using a high-precision scale is only 0.1894 g. This lightweight design significantly enhances wearability, minimizes interference with plant growth, and ensures reliable and accurate long-term monitoring. Owing to its non-invasive characteristics, the sensor offers important advantages for field applications, providing a reliable solution for real-time, dynamic monitoring of physiological features and enabling researchers to gain a comprehensive and accurate understanding of crop growth conditions.

### 2.3 Sample preparation

The tomato experimental samples in this study were seeded on June 7, 2024, in Weifang City, Shandong Province, China. After sufficient growth and management, the samples were transplanted on July 18 to Room 4-27 at the Key Laboratory of Intelligent Agricultural Equipment at Xinjiang Agricultural University for further growth. At the end of the growth period, samples were collected from August 16 to August 17. To ensure the representativeness of the samples, multiple leaf collections were randomly taken from different parts of various plants. During the sampling process, the chlorophyll content of the leaves was first measured using a SPAD meter, followed by spectral data collection to obtain the spectral characteristics of the samples. After sampling, fresh weight measurements of the samples were taken immediately, followed by drying until a constant weight was reached for dry weight measurement to calculate the moisture content and component composition of each sample. The entire experimental process was completed in the laboratory, with no transfer time for the samples, ensuring stable operating conditions and reliability of the data.

### 2.4 Chlorophyll concentration measurement

**Figure 4:**
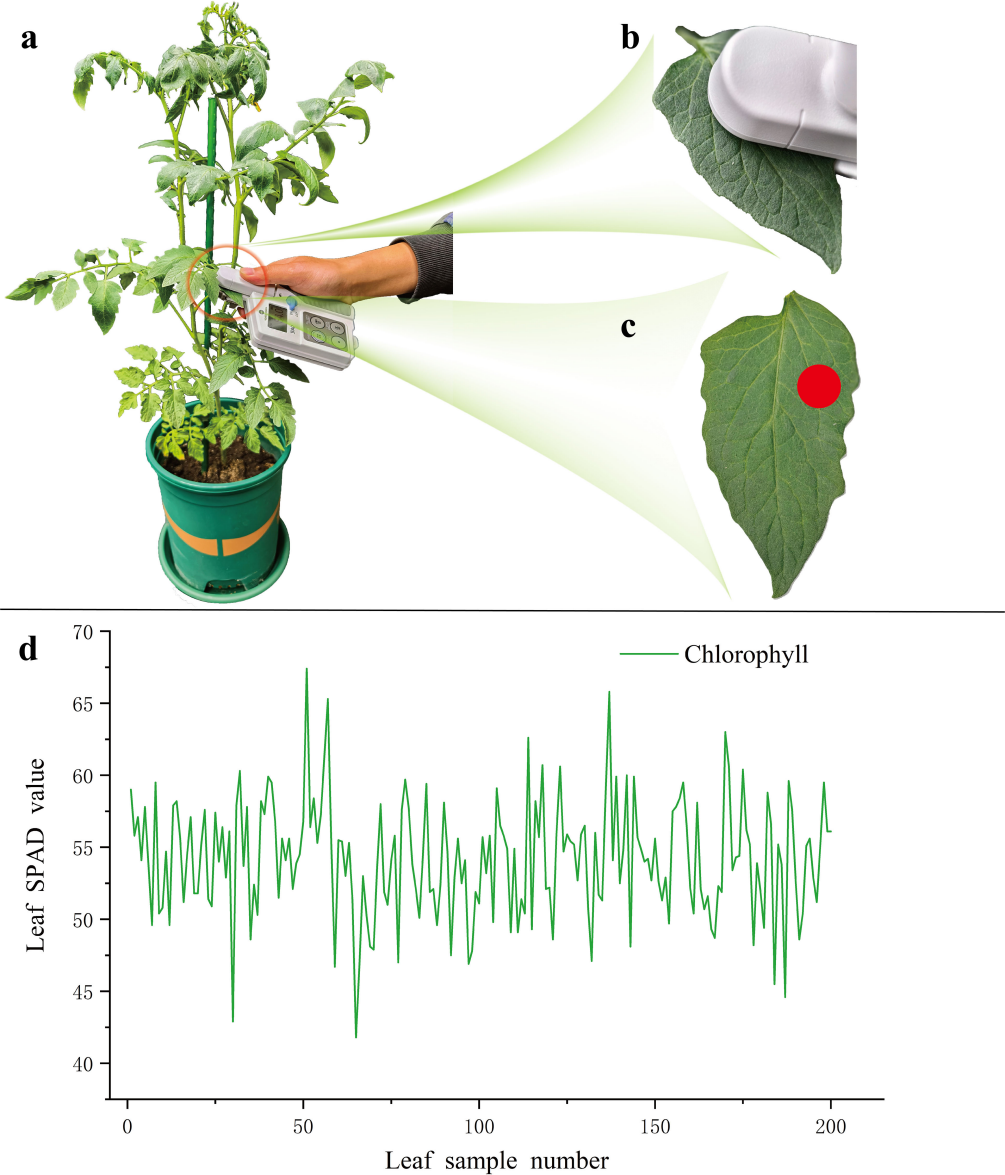
Chlorophyll SPAD measurements. **(a)** Sample selection; **(b)** Chlorophyll measurement; **(c)** Data collection points; **(d)** SPAD data curve.

A total of 80 pots of tomato plants with different growth conditions were selected for SPAD measurement of chlorophyll content, resulting in the collection of 200 samples. During the measurement process, as shown in **Figures 4a, a** leaf without significant pest or disease damage was randomly selected. Care was taken to avoid large veins in the leaf during measurement to prevent measurement errors (Brown et al., 2022). Using the Konica Minolta Chlorophyll Content Meter SPAD-502, as shown in **Figure 4b**, five consecutive measurements were taken at the sampling points indicated in **Figure 4c**, with the average value taken as the final SPAD value for that leaf sampling location, shown in **Figure 4d**.

### 2.5 Leaf spectral data collection

**Figure 5:**
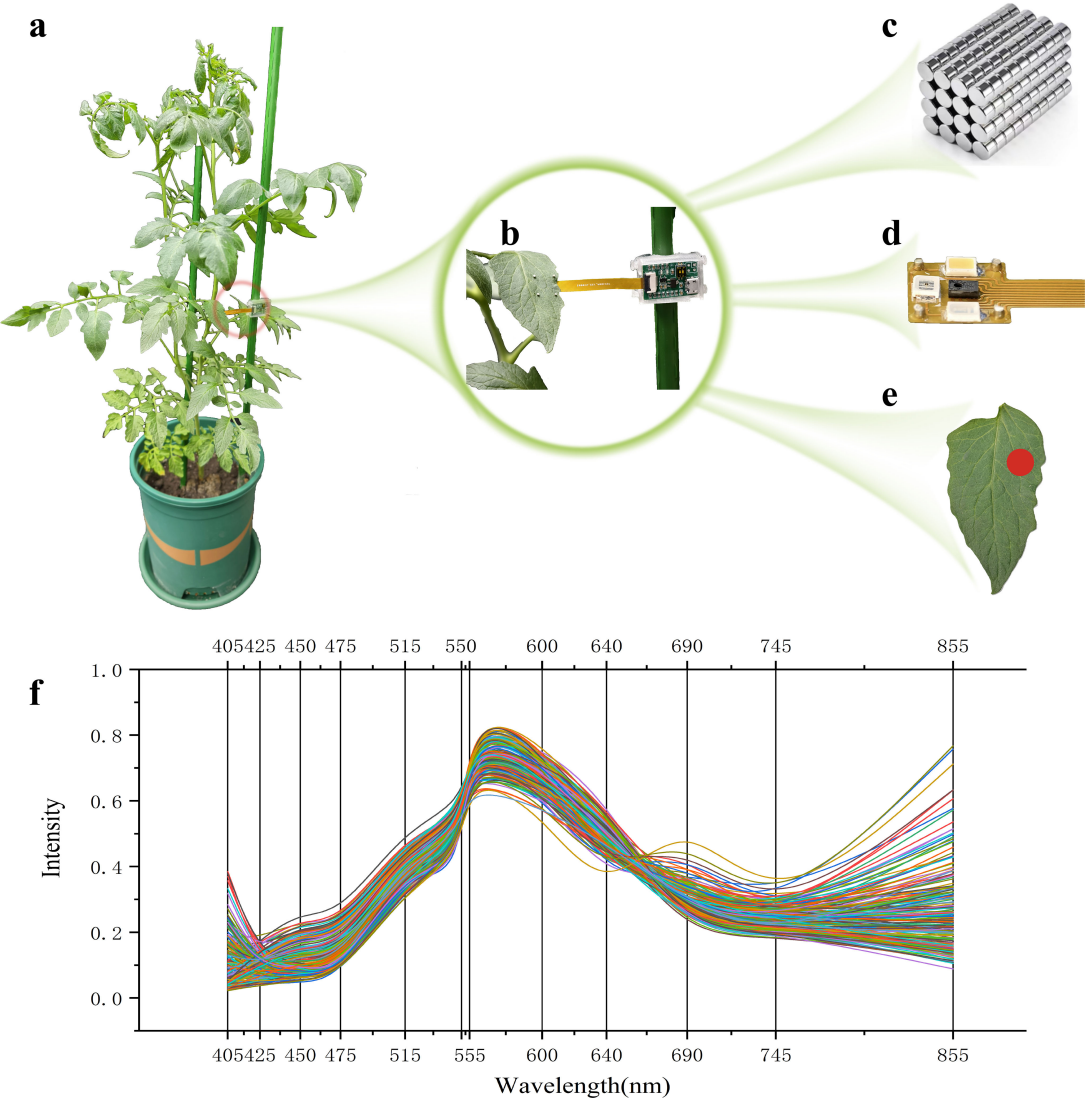
Leaf blade spectral data acquisition. **(a)** Sensor deployment; **(b)** Spectral data collection; **(c)** Magnet; **(d)** Spectral sensor; **(e)** Data collection points; **(f)** Spectral data curve.

After the chlorophyll measurement of the leaves was completed, for the leaves measured by SPAD in **Figure 5a**, the spectral data at the same position as shown in **Figure 5a, e** were collected. The spectral sensor in **Figure 5d** was connected to the small cylindrical powerful magnet in **Figure 5c** through PDMS transparent highly elastic adhesive, and was baked in an oven at a temperature of 80°C for 4 hours to ensure a firm connection. The spectral sensor in **Figure 5b** was placed on the back side of the leaf and fixed in place by using the magnet to complete the installation of the equipment. The spectral data of each sample was collected 10 times, and the average value of each wavelength band was taken as the final spectral data shown in **Figure 5f**.

### 2.6 Leaf water content measurement

**Figure 6:**
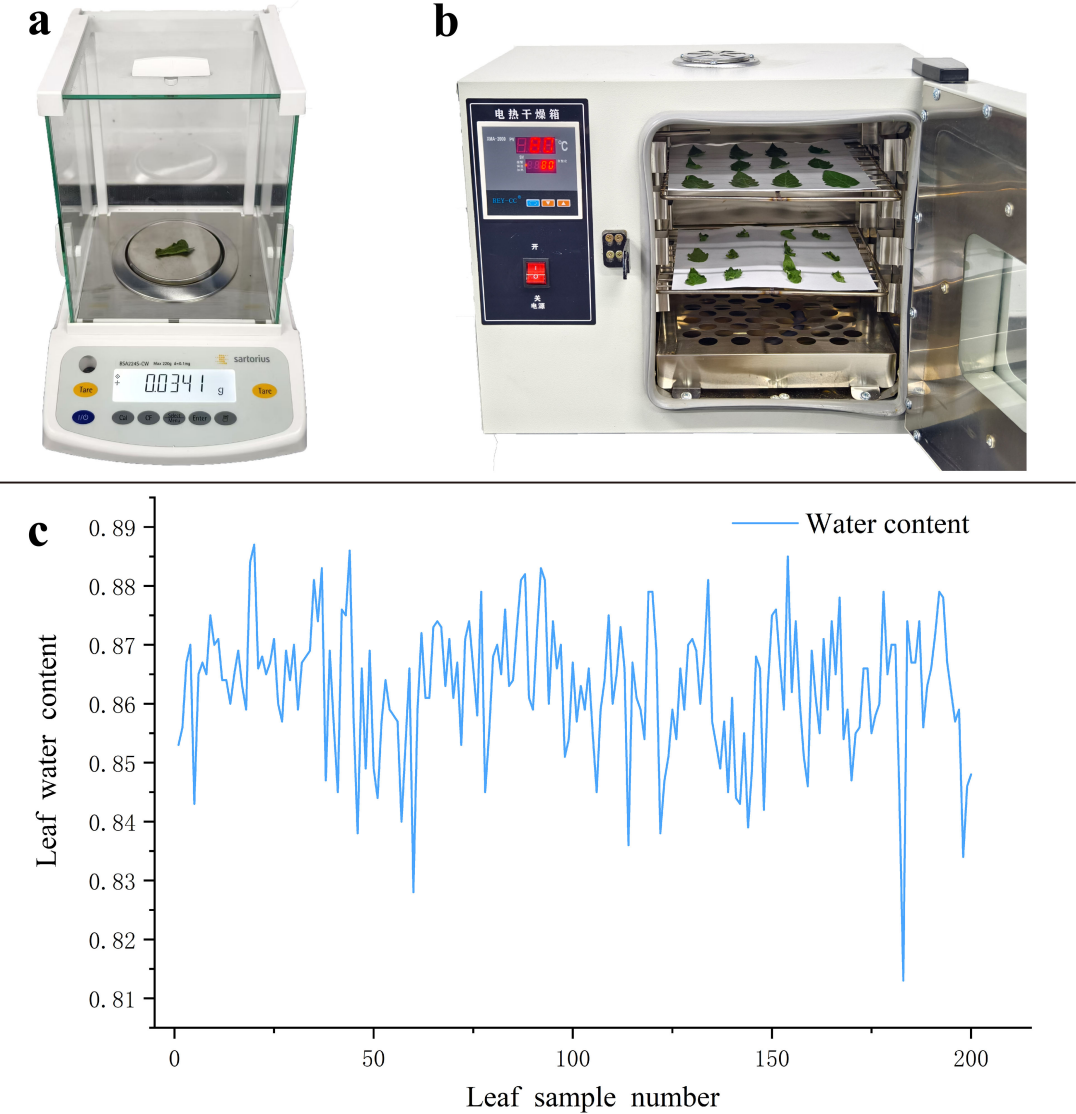
Leaf water content measurement. **(a)** Sample weighing; **(b)** Sample drying treatment; **(c)** Water content data curve.


After completing the spectral data collection, the leaves were placed on the balance shown in **Figure 6a** (accuracy 0.0001g) for fresh weight measurement; at this point, the weight was recorded as fresh weight (FW). After recording the fresh weight, the leaves were immediately placed in the drying oven depicted in **Figure 6b** for drying treatment. The leaves were first blanched in the oven at 105°C for 30 minutes, followed by drying at 80°C. The dry weight (DW) was recorded once three measurements showed no further changes in weight (Han et al., 2022). The formula for calculating leaf water content (LWC) is shown in Equation 1-1, and the distribution of water content for all leaf samples is illustrated in **Figure 6c**.

(1-1) $$\text{LWC}=\frac{\text{FW}-\text{DW}}{\text{FW}}$$

### 2.7 Data processing methods

#### 2.7.1 Data denoising preprocessing

During the collection of spectral data, various noises may arise due to improper equipment installation or environmental lighting conditions. To enhance the accuracy of subsequent modeling and analysis, several preprocessing techniques were employed, including Multiplicative Scatter Correction (MSC), which aims to eliminate scattering effects, making the spectral data of the samples more representative of their true characteristics (Golhani et al., 2019). Standard Normal Variate (SNV) can eliminate the impact of spectral scattering, thereby improving the comparability between different samples (Bao et al., 2012). Wavelet Denoising (WD) utilizes wavelet transformation to decompose and process spectral data, effectively removing noise components through the adjustment of wavelet coefficients (Liu et al., 2024). Smoothing (SMT) reduces random fluctuations in the spectral data to enhance signal stability (Zhang et al., 2022). Normalization (NORM) is applied to standardize the data for easier comparison between different datasets (Shen et al., 2022). Continuum Removal (CR) is used to eliminate baseline drift in the spectral data, thereby improving the accuracy of subsequent analyses (Li et al., 2023b). Derivative Spectroscopy (DS) enhances the discernibility of characteristic signals by calculating the first derivative of the spectrum (Lu et al., 2020). Detrending (DT) is used to remove trend components from the spectral data to ensure the purity of the data signal (Li et al., 2024). Various effective denoising techniques were applied to preprocess the spectral data.

#### 2.7.2 Machine learning ensemble algorithms

In the analysis of spectral data for predicting leaf SPAD values and water content, this study employed fourteen regression algorithms for comprehensive evaluation. These algorithms include Linear Regression (LR), which is suitable for linear relationships and can quickly yield predictive results that are easy to interpret (Zhai et al., 2024); Ridge Regression (RR), which effectively handles multicollinearity and reduces model complexity, particularly suited for high-dimensional data (Li et al., 2023c); Huber Regression (HR), known for its robustness against outliers, making it appropriate for datasets with a few outliers (Wu et al., 2023); K-Nearest Neighbors Regression (KNN), whose simple and intuitive nature does not require assumptions about data distribution, allowing it to capture complex nonlinear relationships (Hou et al., 2022); Random Forest Regression (RFR), which adapts to the analysis of complex nonlinear relationships by processing high-dimensional data and reducing overfitting (Yuan et al., 2021); AdaBoost Regression (ABR), which improves prediction accuracy by combining multiple weak learners through weighting, demonstrating high robustness (Wang et al., 2021a); Gradient Boosting Regression (GBR), which effectively captures complex nonlinear relationships with good predictive performance, suitable for handling large-scale data (Wu et al., 2023); Bagging Regression (BR), which enhances robustness by reducing model variance, appropriate for irregular data distributions (Li et al., 2023a); Gaussian Process Regression (GPR), which provides uncertainty estimates and is particularly suitable for small sample data while being adaptable to different function shapes (Adeluyi et al., 2021); Partial Least Squares Regression (PLSR), which effectively extracts information when faced with highly correlated independent variables, particularly suited for high-dimensional data (Zhang et al., 2023); Support Vector Regression (SVR), capable of handling complex nonlinear relationships by finding the best hyperplane (Wang et al., 2021b); Transformed Target Regression (TTR), which improves the model’s linear characteristics by transforming the target variable to enhance predictive performance; Lasso Regression (LAR), which uses L1 regularization to select features and reduce model complexity, suitable for producing sparse solutions (Yang et al., 2023); and ElasticNet Regression (ENR), which combines L1 and L2 regularization to provide effective predictions in scenarios with multicollinearity (Fei et al., 2023). In the context of predicting leaf SPAD values and water content, these algorithms each have their strengths, offering diverse options for achieving precise predictions.

## 3 Results

### 3.1 Analysis of raw spectral data

**Figure 7:**
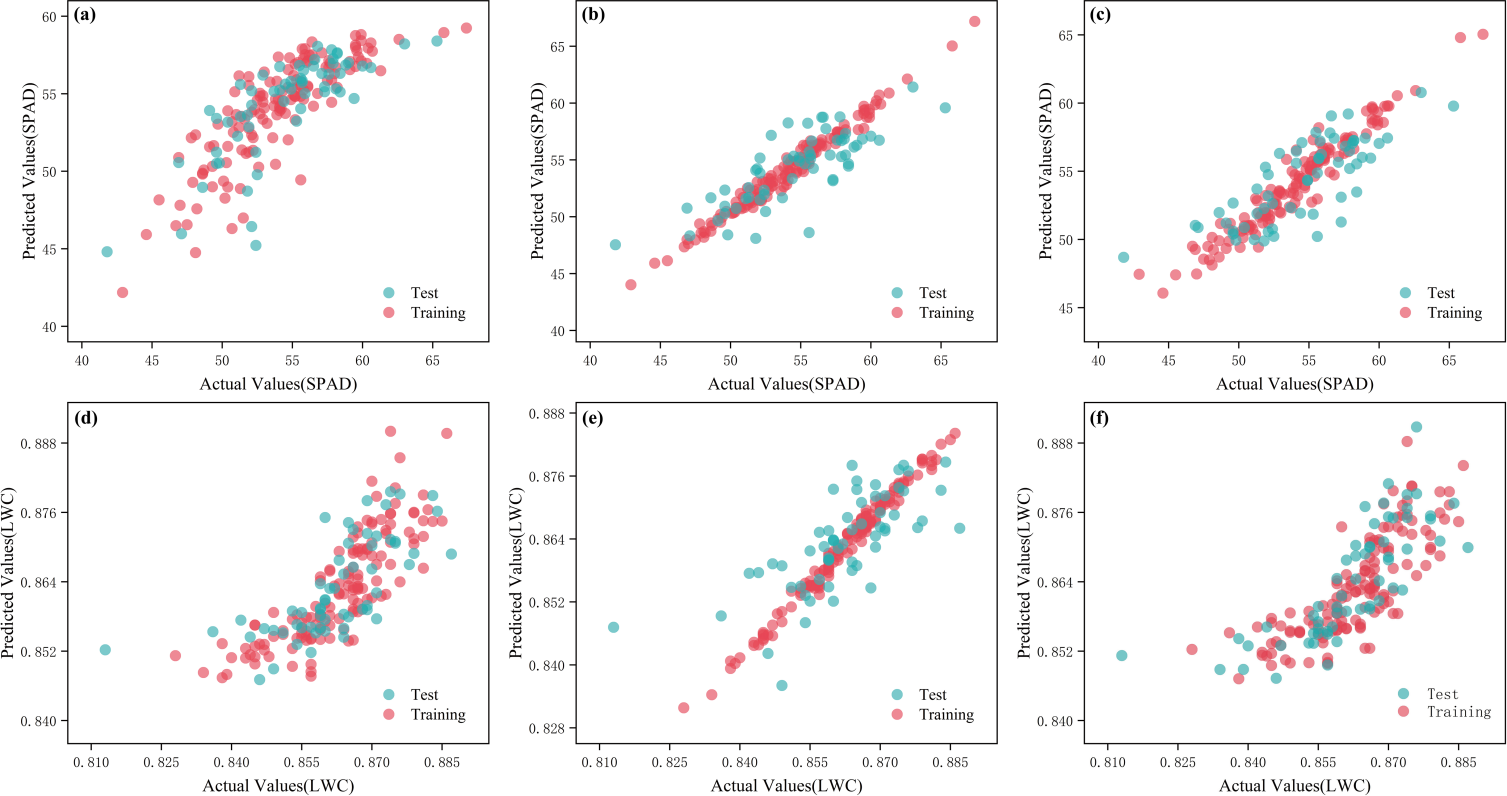
Measured and predicted values of different models of raw spectra. **(a)** LAR-SPAD; **(b)** GBR-SPAD; **(c)** BR-SPAD; **(d)** RR-LWC; **(e)** GBR-LWC; **(f)** TTR-LWC.

Different model frameworks applied to measured data may yield varying predictive results. To achieve the best predictive model, multiple distinct model frameworks were selected for comparative analysis. The data was divided with 70% allocated to the training set and 30% to the prediction set. Ultimately, the model with the best performance was determined based on the coefficient of determination and root mean square error, selected as the final predictive model. Machine learning was utilized to perform regression analysis on the SPAD values and water content of tomato leaves using the raw spectral data. The results are shown in **Table 1**. The analysis found that for chlorophyll across all spectral wavelength combinations, LAR, GBR, and BR regression algorithms all performed well in predicting SPAD, with GBR demonstrating the best predictive capability. In the training set, GBR had an R_c_² of 0.9741 and RMSE_c_ of 0.6526; in the testing set, GBR’s R_p_² was 0.6128 and RMSE_p_ was 2.5606. For water content across all spectral wavelength combinations, RR, GBR, and TTR regression algorithms also performed well in predicting water content, with GBR again yielding the best results. In the training set, GBR had an R_c_² of 0.9850 and RMSE_c_ of 0.0014; in the testing set, GBR’s R_p_² was 0.4946 and RMSE_p_ was 0.0087. The predictive models for leaf water content and chlorophyll are illustrated in **Figure 7**.

**Table 1:** Raw spectral analysis.

Target	Prediction method	Wavelength (nm)	Training set		Test set	
			R_c_^2^	RMSE_c_	R_p_^2^	RMSE_p_
SPAD	KNN	640	0.6318	2.4590	0.5240	2.8390
	RR	All	0.6917	2.2504	0.5662	2.7103
	LAR	All	0.6882	2.2630	0.5909	2.6318
	RFR	All	0.9388	1.0025	0.5630	2.7201
	ABR	All	0.8252	1.6945	0.5321	2.8147
	GBR	All	0.9741	0.6526	0.6128	2.5606
	BR	All	0.9234	1.1215	0.5881	2.6410
	TTR	All	0.6917	2.2504	0.5662	2.7103
LWC	KNN	All	0.7656	0.0054	0.3624	0.0097
	RR	All	0.6725	0.0064	0.4857	0.0087
	RFR	All	0.9594	0.0023	0.4644	0.0089
	ABR	All	0.8706	0.0040	0.4033	0.0094
	GBR	All	0.9850	0.0014	0.4946	0.0087
	BR	All	0.9450	0.0026	0.4514	0.0090
	ENR	All	0.6038	0.0070	0.4153	0.0093
	TTR	All	0.6320	0.0066	0.5728	0.0084

**Figure 8:**
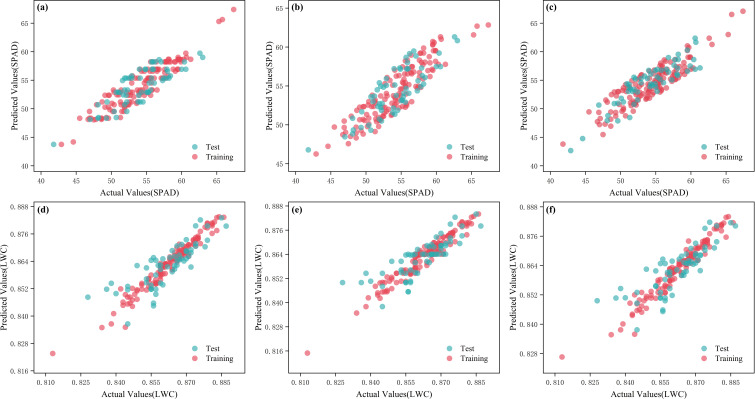
Measured and predicted values of different models of the original relative spectra. **(a)** ABR-SPAD; **(b)** HR-SPAD; **(c)** GPR-SPAD; **(d)** RFR-LWC; **(e)** GBR-LWC; **(f)** BR-LWC.

The Clear band of the AS7343 spectral sensor positively impacts measurement accuracy and data comparability, helping to accurately identify background light and enhancing the signal-to-noise ratio. It can serve as a calibration baseline for other bands, effectively compensating for variations in ambient light, sensor characteristics, and object reflectance, while measuring the overall intensity of incident light. By dividing the intensity of the Clear band by the intensity of other bands, the original relative intensity spectrum is obtained. This method effectively standardizes the data, mitigating the influence of different lighting conditions on measurement results, thereby enhancing consistency. Machine learning was applied to the original relative spectral data to perform regression analysis on the SPAD values and water content of tomato leaves, as shown in **Table 2**. The analysis found that for chlorophyll at 640 nm, ABR, HR, and GPR regression algorithms all performed well in predicting SPAD, with GPR demonstrating the best performance. In the training set, GPR had an R_c_² of 0.8027 and RMSE_c_ of 1.8176; in the testing set, GPR’s R_p_² was 0.7784 and RMSE_p_ was 1.9038. For water content at the spectral 855 nm band, RFR, GBR, and BR regression algorithms also performed well in predicting water content, with GBR again yielding the best results. In the training set, GBR had an R_c_² of 0.9401 and RMSE_c_ of 0.0028; in the testing set, GBR’s R_p_² was 0.6667 and RMSE_p_ was 0.0067. The predictive models for leaf water content and chlorophyll based on the original relative spectral data are shown in **Figure 8**.

**Table 2:** Analysis of raw relative spectral data.

Target	Prediction method	Wavelength (nm)	Training set		Test set	
			R_c_^2^	RMSE_c_	R_p_^2^	RMSE_p_
SPAD	LR	640	0.8057	1.8435	0.6730	2.1840
	KNN	640	0.8346	1.7012	0.6441	2.2785
	SVR	640	0.7408	2.1294	0.6415	2.2869
	RFR	All	0.9665	0.7652	0.6627	2.2181
	ABR	640	0.8547	1.5941	0.6755	2.1756
	GBR	All	0.9897	0.4252	0.6504	2.2583
	BR	All	0.9567	0.8705	0.6637	2.2148
	PLSR	640	0.8057	1.8435	0.6730	2.1840
	HR	640	0.8041	1.8514	0.6762	2.1734
	GPR	640	0.8027	1.8176	0.7784	1.9038
	TTR	640	0.8057	1.8435	0.6730	2.1840
LWC	LR	All	0.7913	0.0052	0.5881	0.0075
	KNN	855	0.8208	0.0048	0.6345	0.0070
	RR	All	0.7087	0.0062	0.5801	0.0075
	RFR	855	0.9518	0.0025	0.6604	0.0068
	ABR	855	0.8450	0.0045	0.6177	0.0072
	GBR	855	0.9401	0.0028	0.6667	0.0067
	BR	855	0.9440	0.0027	0.6554	0.0068
	PLSR	855	0.7614	0.0056	0.5868	0.0075
	HR	All	0.7750	0.0054	0.6130	0.0072
	GPR	855	0.7790	0.0054	0.6122	0.0073
	TTR	All	0.7913	0.0052	0.5881	0.0075

### 3.2 Analysis of spectral data denoising

**Figure 9:**
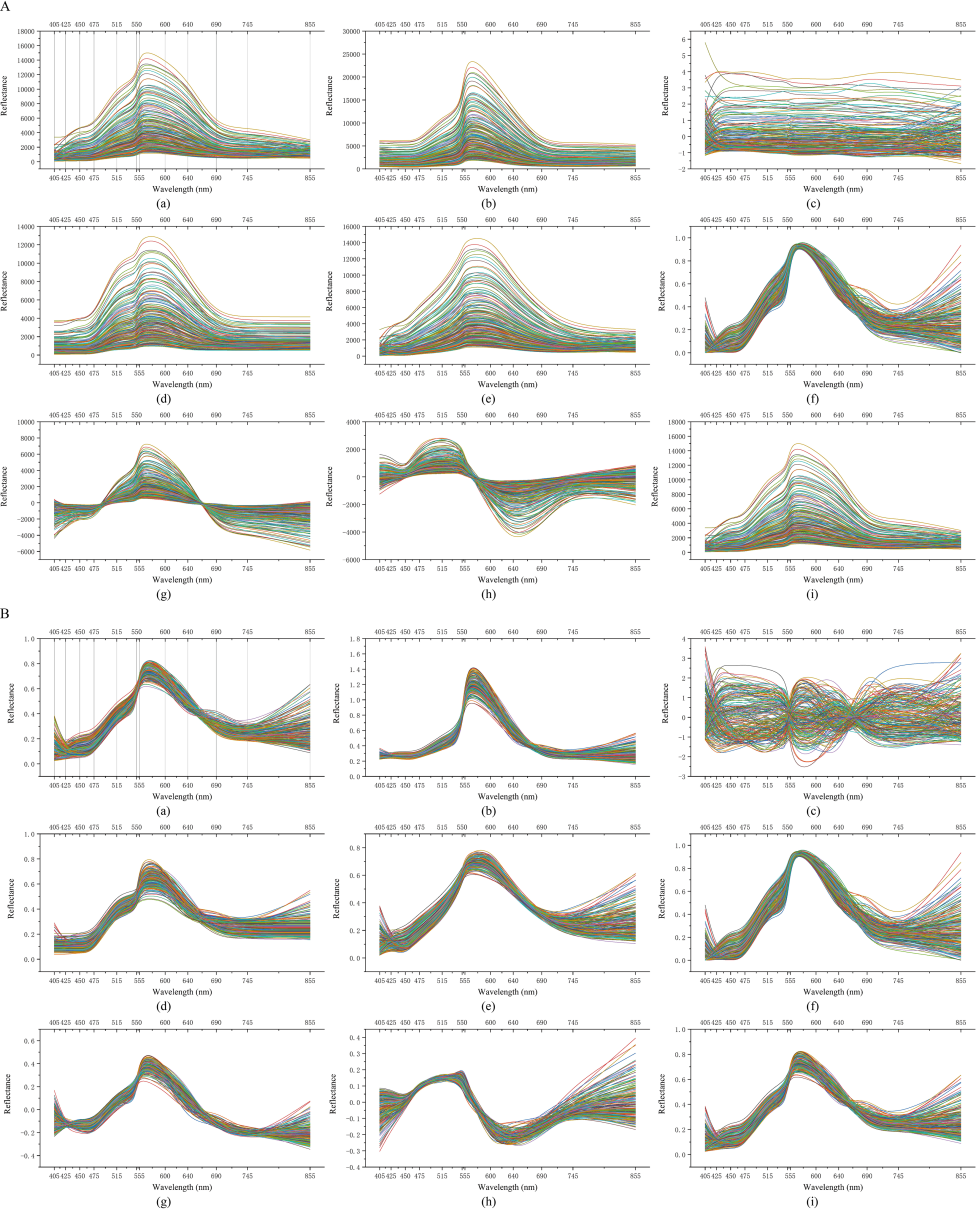
Noise reduction processing of spectral data. **(A, B)**: (a) raw; (b) MSC; (c) SNV; (d) WD; (e) SMT; (f) NORM; (g) CR; (h) DS; (i) DT.

To effectively extract information from spectral data, various spectral preprocessing methods were employed to achieve data denoising. Spectral data is often affected by noise due to factors such as improper equipment installation and ambient light, which reduces the clarity and interpretability of the signals. Therefore, implementing diverse denoising strategies is crucial for improving spectral quality. Through systematic denoising treatment, interference signals were suppressed, leading to enhanced signal strength and interpretative capability of the denoised raw spectral and original relative spectral data. This allowed the effective information within the spectra to become more prominent, aiding in the enhancement of spectral features’ significance and reliability, while demonstrating good stability and robustness under multiple experimental conditions. As shown in **Figures 9A, B**, the spectral plots of the raw spectral data and original relative spectral data exhibit differences in clarity and detail retention before and after denoising.

**Figure 10:**
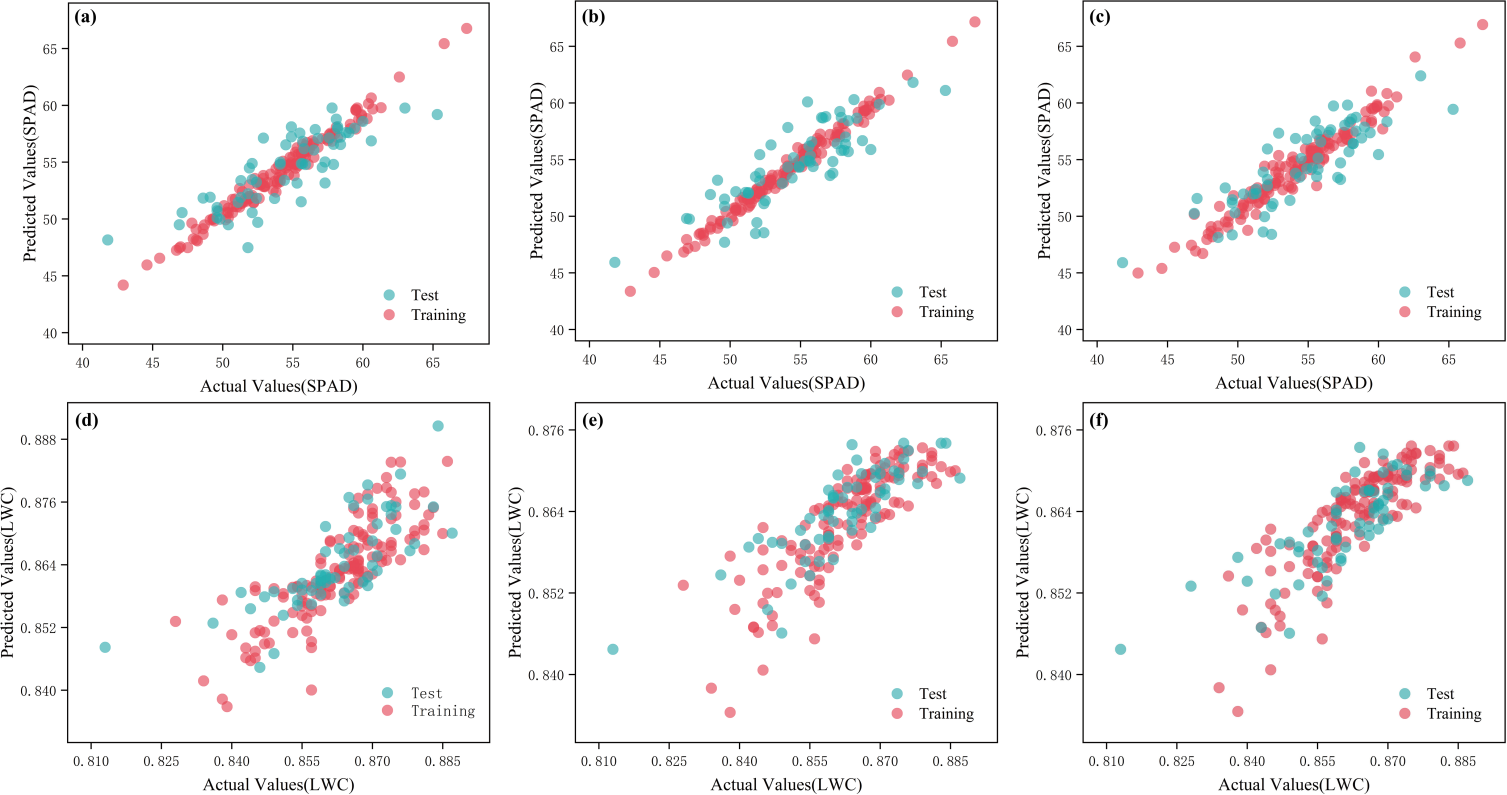
Measured and predicted values for different models of the original noise reduction spectra. **(a)** MSC-GBR-SPAD; **(b)** NORM-GBR-SPAD; **(c)** NORM-BR-SPAD; **(d)** WD-TTR-LWC; **(e)** NORM-RR-LWC; **(f)** NORM-TTR-LWC.

To assess the effectiveness of the denoising preprocessing methods applied to the raw spectral data, three regression algorithms that exhibited the highest accuracy in predicting SPAD values were selected: LAR, GBR, and BR. Additionally, for predicting leaf water content, the best-performing three regression algorithms were RR, GBR, and TTR. The study will conduct a detailed analysis and prediction for each wavelength and all wavelength combinations to systematically evaluate the impact of the preprocessed spectral data on the predictive models. The analysis results are shown in **Table 3**. In the SPAD prediction section, it was observed that four denoising methods MSC, NORM, CR, and DS all showed a trend of improved prediction accuracy. Notably, MSC and NORM had the most significant effects, with determination coefficients R_p_² of 0.6782, 0.7070, and 0.6832 on the test set, demonstrating the positive impact of preprocessing on SPAD data. However, under the NORM denoising condition, Lasso regression failed to successfully predict SPAD values. In the prediction analysis of leaf water content, the four denoising methods SNV, WD, NORM, and DS also exhibited a trend of enhanced prediction accuracy. Among them, the improvements from WD and NORM were particularly noticeable, with determination coefficients R_p_² of 0.5456, 0.5780, and 0.6227 on the test set, indicating that denoising treatment played a decisive role in enhancing model performance. The predictive models for leaf SPAD and leaf water content based on the original denoised spectral data are shown in **Figure 10**.

**Table 3:** Noise reduction analysis of raw spectral data.

Noise reduction methods	Prediction method	Target	Wavelength (nm)	Training set		Test set	
				R_c_^2^	RMSE_c_	R_p_^2^	RMSE_p_
MSC	LAR	SPAD	All	0.6867	2.2683	0.5954	2.6176
	GBR		All	0.9722	0.6760	0.6782	2.3342
	BR		All	0.9282	1.0862	0.6026	2.5940
	RR	LWC	All	0.6725	0.0064	0.4857	0.0087
	GBR		All	0.9814	0.0015	0.4808	0.0088
	TTR		All	0.6725	0.0064	0.4857	0.0087
SNV	LAR	SPAD	640	0.3567	3.2506	0.3445	303317
	GBR		All	0.9741	0.6526	0.6128	2.5606
	BR		All	0.9234	1.1215	0.5881	2.6410
	RR	LWC	All	0.6459	0.0067	0.5073	0.0085
	GBR		All	0.9850	0.0014	0.4946	0.0087
	TTR		All	0.6725	0.0064	0.4857	0.0087
WD	LAR	SPAD	All	0.6355	2.4468	0.5727	2.6898
	GBR		All	0.9679	0.7266	0.5472	2.7689
	BR		All	0.9050	1.2489	0.5543	2.7471
	RR	LWC	All	0.6810	0.0063	0.5456	0.0082
	GBR		All	0.9819	0.0015	0.4397	0.0091
	TTR		All	0.6810	0.0063	0.5456	0.0082
SMT	LAR	SPAD	All	0.6831	2.2815	0.6125	2.5614
	GBR		All	0.9789	0.5888	0.5618	2.7241
	BR		All	0.9078	1.2304	0.5350	2.8059
	RR	LWC	All	0.6725	0.0064	0.4857	0.0087
	GBR		All	0.9852	0.0014	0.4610	0.0089
	TTR		All	0.6725	0.0064	0.4857	0.0087
NORM	LAR	SPAD	–	–	–	–	–
	GBR		All	0.9863	0.4743	0.7070	2.2275
	BR		All	0.9466	0.9364	0.6832	2.3160
	RR	LWC	855	0.6892	0.0061	0.5630	0.0083
	GBR		855	0.9256	0.0030	0.4772	0.0088
	TTR		855	0.7045	0.0061	0.6227	0.0075
CR	LAR	SPAD	All	0.6517	2.3916	0.5053	2.8944
	GBR		All	0.9838	0.5163	0.5991	2.6055
	BR		All	0.9424	0.9729	0.5680	2.7047
	RR	LWC	All	0.6282	0.0068	0.3979	0.0094
	GBR		555	0.8962	0.0036	0.4075	0.0094
	TTR		All	0.6410	0.0067	0.3890	0.0095
DS	LAR	SPAD	All	0.6447	2.4156	0.5209	2.8483
	GBR		All	0.9833	0.5241	0.6369	2.4796
	BR		All	0.9278	1.0892	0.5806	2.6648
	RR	LWC	855	0.6010	0.0071	0.4932	0.0087
	GBR		All	0.9799	0.0016	0.5115	0.0085
	TTR		855	0.6010	0.0071	0.4932	0.0087
DT	LAR	SPAD	All	0.6882	2.2630	0.5909	2.6318
	GBR		All	0.9741	0.6526	0.6128	2.5606
	BR		All	0.9234	1.1215	0.5881	2.6410
	RR	LWC	All	0.6725	0.0064	0.4857	0.0087
	GBR		All	0.9850	0.0014	0.4946	0.0087
	TTR		All	0.6725	0.0064	0.4857	0.0087

**Figure 11:**
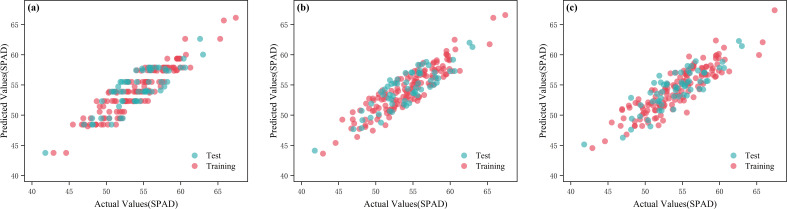
Measured and predicted values for different models of the original relative noise reduction spectra. **(a)** MSC-ABR-SPAD; **(b)** MSC-GPR-SPAD; **(c)** CR-GPR-SPAD.

To evaluate the effectiveness of the denoising preprocessing methods applied to the original relative spectral data, three regression algorithms that exhibited the highest accuracy in predicting SPAD values in the original relative spectral data were selected: ABR, HR, and GPR; at the same time, the three best-performing regression algorithms for predicting leaf water content: RFR, GBR, and BR. The study will conduct detailed analyses and predictions for each wavelength and all wavelength combinations to systematically assess the impact of the preprocessed spectral data on the predictive models. The analysis results are shown in **Table 4**. In the SPAD prediction section, it was observed that the two denoising methods, MSC and CR, both demonstrated a trend of improving prediction accuracy. Their determination coefficients R_p_^2^ on the test set were 0.7113, 0.7155, and 0.7128, indicating a positive impact of preprocessing on SPAD data. In the prediction analysis of leaf water content, the best prediction effect was the same as the prediction performance of the original relative spectral data, with determination coefficients R_p_^2^ of 0.6604, 0.6667, and 0.6554 on the test set. The effect of the leaf SPAD prediction model based on the original relative denoised spectral data is shown in **Figure 11**.

**Table 4:** Noise reduction analysis of raw relative spectral data.

Noise reduction methods	Prediction method	Target	Wavelength (nm)	Training set		Test set	
				R_c_^2^	RMSE_c_	R_p_^2^	RMSE_p_
MSC	ABR	SPAD	640	0.8462	1.6403	0.7113	2.0524
	HR		640	0.8034	1.8544	0.6817	2.1548
	GPR		640	0.8261	1.7444	0.7155	2.0374
	RFR	LWC	855	0.9509	0.0025	0.6116	0.0073
	GBR		855	0.9383	0.0028	0.6216	0.0072
	BR		855	0.9430	0.0027	0.6100	0.0073
SNV	ABR	SPAD	640	0.8547	1.5941	0.6755	2.1756
	HR		640	0.8041	1.8512	0.6762	2.1734
	GPR		640	0.8371	1.6881	0.7083	2.0629
	RFR	LWC	855	0.9518	0.0025	0.6604	0.0068
	GBR		855	0.9401	0.0028	0.6667	0.0067
	BR		855	0.9440	0.0027	0.6554	0.0068
WD	ABR	SPAD	All	0.8475	1.6334	0.4615	2.8027
	HR		All	0.7322	2.1645	0.5348	2.6051
	GPR		855	0.5128	2.9194	0.4249	2.8964
	RFR	LWC	855	0.9470	0.0026	0.5808	0.0075
	GBR		855	0.9394	0.0028	0.6125	0.0072
	BR		855	0.9322	0.0030	0.5519	0.0078
SMT	ABR	SPAD	All	0.8996	1.3254	0.6288	2.3272
	HR		All	0.8060	1.8420	0.6646	2.2121
	GPR		745	0.6601	2.4386	0.5583	2.5384
	RFR	LWC	855	0.9566	0.0024	0.5369	0.0079
	GBR		855	0.9483	0.0026	0.5731	0.0076
	BR		855	0.9474	0.0026	0.5243	0.0080
NORM	ABR	SPAD	All	0.8937	1.3638	0.5590	2.5364
	HR		All	0.7692	2.0093	0.6130	2.3761
	GPR		640	0.6767	2.3784	0.4456	2.8439
	RFR	LWC	All	0.9602	0.0023	0.5201	0.0081
	GBR		All	0.9844	0.0014	0.5202	0.0081
	BR		All	0.9502	0.0025	0.5019	0.0082
CR	ABR	SPAD	640	0.8245	1.7521	0.6829	2.1508
	HR		640	0.7604	2.0474	0.6835	2.1486
	GPR		640	0.7868	1.9312	0.7128	2.0468
	RFR	LWC	All	0.9573	0.0024	0.5575	0.0077
	GBR		All	0.9909	0.0011	0.5346	0.0079
	BR		All	0.9426	0.0027	0.5669	0.0077
DS	ABR	SPAD	All	0.9032	1.3016	0.6137	2.3740
	HR		All	0.8030	1.8566	0.6688	2.1981
	GPR		690	0.7417	2.1259	0.5423	2.5840
	RFR	LWC	All	0.9610	0.0023	0.5219	0.0081
	GBR		All	0.9876	0.0013	0.5492	0.0078
	BR		All	0.9503	0.0025	0.4928	0.0083
DT	ABR	SPAD	640	0.8547	1.5941	0.6755	2.1756
	HR		640	0.8041	1.8514	0.6762	2.1734
	GPR		640	0.8260	1.7445	0.7112	2.0525
	RFR	LWC	855	0.9518	0.0025	0.6604	0.0068
	GBR		855	0.9401	0.0028	0.6667	0.0067
	BR		855	0.9440	0.0027	0.6554	0.0068

### 3.3 Multi-model fusion analysis

**Figure 12:**
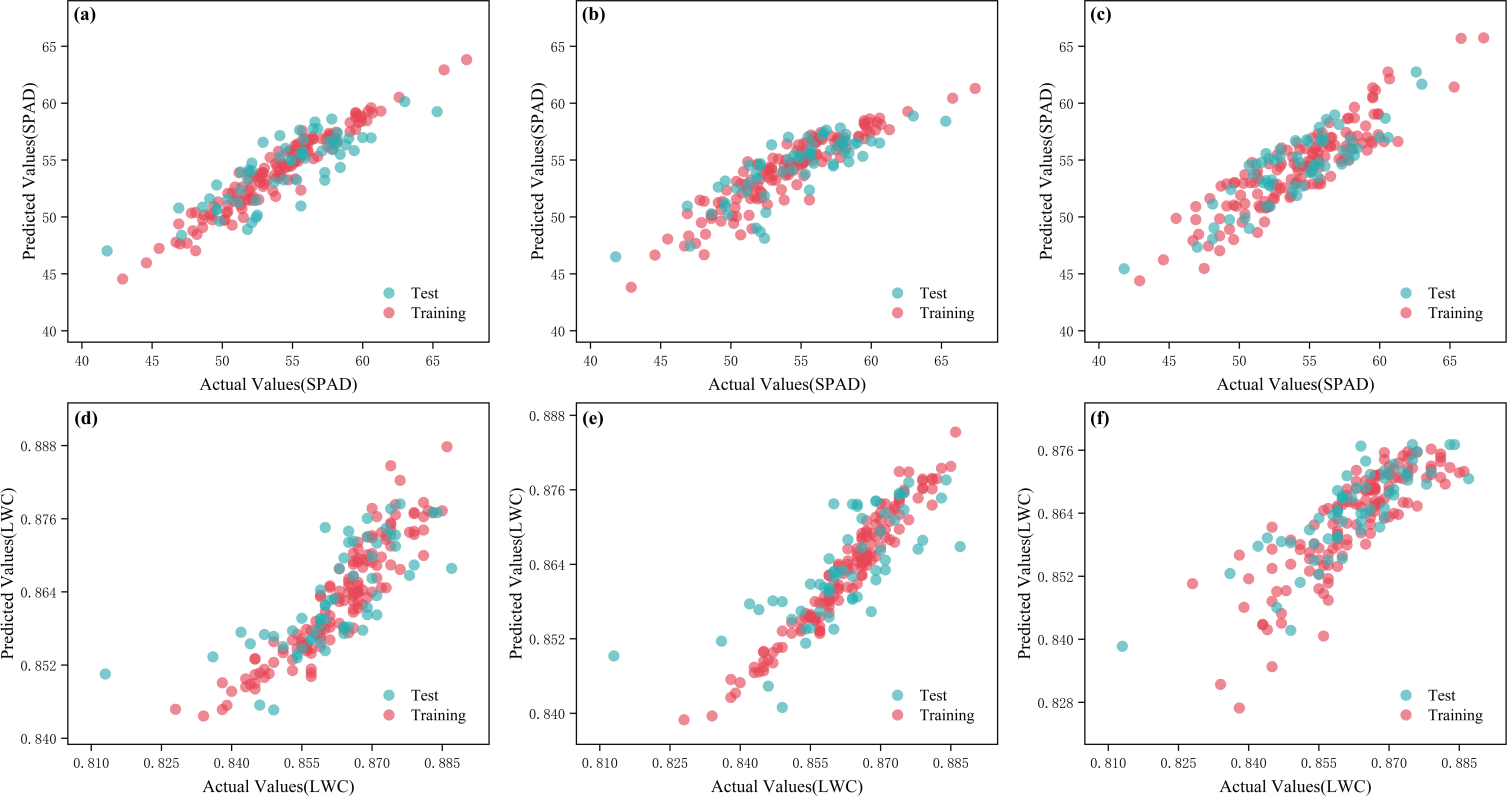
Measured and predicted values of multi-model fusion analysis of spectral data. **(a)** Raw-VR-LAR-GBR-BR-SPAD; **(b)** Raw-SR-LAR-GBR-BR-SPAD; **(c)** Relative-SR-ABR-HR-GPR-SPAD; **(d)** Raw-VR-RR-GBR-TTR-LWC; **(e)** Raw-SR-RR-GBR-TTR-LWC; **(f)** Raw-NORM-SR-RR-TTR-LWC.

A multi-model fusion analysis method was employed to enhance the prediction accuracy of leaf water content and chlorophyll content. Two fusion models, VotingRegressor and StackingRegressor, were selected to combine the original spectral data, original relative spectral data, and the denoised spectral data that had high correlations with leaf water content and chlorophyll through relevant regression models, aiming for a more accurate prediction of the target variables. VotingRegressor combines the advantages of various models by performing weighted voting on the outputs of multiple base regression models, reducing potential biases from individual models and enhancing the overall model’s robustness. StackingRegressor, on the other hand, constructs a multi-layer model where the prediction results of different regression models are used as new feature inputs to a higher-level model, thus achieving more complex feature combinations and more accurate regression predictions. The analysis results are shown in **Table 5**. For the original spectral data, in the prediction of SPAD, the VR and SR fusion methods used LAR, GBR, and BR as base learning models, effectively predicting SPAD through all combinations of spectra, leading to an improvement in the best prediction performance of the original spectrum, with determination coefficients R_p_^2^ of 0.6590 and 0.6399 on the test set, respectively. Simultaneously, the VR and SR fusion methods used RR, GBR, and TTR as base learning models, effectively predicting leaf water content through all combinations of spectra and the 855 nm spectral band, leading to an improvement in the best prediction performance of the original spectrum, with determination coefficients R_p_^2^ of 0.5254 and 0.5246 on the test set, respectively. Regarding the original denoised spectral data, under NORM denoising, the SR fusion method used RR and TTR as base learning models, effectively predicting leaf water content through spectral data from the 855 nm band, with an improvement in the best prediction performance of the original denoised spectrum, resulting in a determination coefficient R_p_^2^ of 0.6286 on the test set. For the relative spectral data, the SR fusion method used ABR, HR, and GPR as base learning models, effectively predicting leaf SPAD through spectral data from the 640 nm band, with an improvement in the best prediction performance of the relative spectrum, leading to a determination coefficient R_p_^2^ of 0.7114 on the test set. The use of relative spectral denoised data did not show significant improvement in the prediction results for leaf water content and SPAD. The effects of the multi-model fusion method for spectral data on the prediction models of leaf water content and SPAD are shown in **Figure 12**.

**Table 5:** Multi-model fusion analysis of spectral data.

Spectral type	Fusion method	Regression model	Target	Wavelength (nm)	Training set		Test set	
					R_c_^2^	RMSE_c_	R_p_^2^	RMSE_p_
Raw	VR	LAR-GBR-BR	SPAD	All	0.9161	1.1742	0.6590	2.4030
	SR				0.8201	1.7188	0.6399	2.4694
	VR	RR-GBR-TTR	LWC	All	0.8335	0.0046	0.5254	0.0084
	SR				0.9336	0.0029	0.5264	0.0084
Raw-MSC	VR	LAR-GBR-BR	SPAD	All	0.9152	1.1802	0.6873	2.3009
	SR				0.8230	1.7048	0.6690	2.3675
Raw-NORM	VR				0.8455	1.5927	0.6304	2.5016
	SR				0.9603	0.8078	0.7010	2.2502
Raw-WD	VR	RR-TTR	LWC	All	0.6810	0.0063	0.5456	0.0082
	SR				0.6757	0.0064	0.5352	0.0083
Raw-NORM	VR			855	0.6990	0.0061	0.6039	0.0077
	SR				0.7032	0.0061	0.6286	0.0074
Relative	VR	ABR-HR-GPR	SPAD	640	0.8392	1.6773	0.6966	2.1038
	SR				0.7972	1.8837	0.7114	2.0518
	VR	RFR-GBR-BR	LWC	855	0.9524	0.0025	0.6647	0.0067
	SR				0.9272	0.0031	0.6601	0.0068
Relative-MSC	VR	ABR-HR-GPR	SPAD	640	0.8354	1.6968	0.7120	2.0498
	SR				0.8035	1.8541	0.7048	2.0751
Relative-CR	VR				0.8021	1.8608	0.7050	2.0747
	SR				0.7848	1.9403	0.7145	2.0409
Relative-SNV	VR	FRF-GBR-BR	LWC	855	0.9524	0.0025	0.6647	0.0067
	SR				0.9272	0.0031	0.6601	0.0068
Relative-DT	VR				0.9524	0.0025	0.6647	0.0067
	SR				0.9272	0.0031	0.6601	0.0068

## 4 Discussion

The flexible wearable spectral sensor developed in this study offers significant advantages for monitoring the physiological characteristics of plant leaves, particularly through non-destructive and real-time *in situ* monitoring. The sensor effectively measures SPAD values and water content, providing superior long-term monitoring outcomes compared to traditional monitoring methods. The results indicate that this sensor is well-suited for real-time acquisition of plant physiological information, providing new technical support for agricultural management.

The design of the sensor features lightweight construction, ease of operation, and flexible arrangement, enabling non-invasively attachment to the underside of plant leaves. This characteristic significantly reduces the impact on plant growth, especially during critical physiological processes such as photosynthesis and transpiration. By fixing the sensor with a magnetic attachment, the stability and reliability of long-term data collection are ensured. Experimental results indicate significant performance differences among various regression models for predicting SPAD values and water content, with GRP and GBR performing particularly well, especially after data preprocessing, which greatly enhanced their prediction accuracy. These results demonstrate the effectiveness of the integrated sensor design and lay a solid foundation for advancing field agriculture monitoring.

In terms of data processing, this study employed various denoising preprocessing methods on the collected spectral data to enhance data quality. Traditional spectral analysis is often affected by improper equipment installation and environmental interference, severely impacting data accuracy. By introducing noise reduction strategies such as multivariate scatter correction and standard normal variate transformation, the model’s predictive capability was significantly enhanced. This not only improved data reliability but also provided necessary adaptability for data collection in complex environments.

In selecting machine learning models, this study conducted extensive analyses of various regression algorithms, including LR, RF, and GBR. The use of multi-model fusion methods further enhanced the predictive performance of leaf SPAD values and water content. Particularly when employing the VR and SR fusion models, the prediction accuracy for leaf physiological indicators was effectively enhanced. The comprehensive use of these algorithms not only improved predictive capability but also reduced dependency on a single dataset, enabling better performance across a wider range of application scenarios.

The *in situ* tracking technology of wearable flexible spectral sensors offers a new pathway for precision management in modern agriculture. By monitoring the physiological characteristics of plant leaves in real-time, agricultural producers can promptly acquire the growth status of crops, enabling the formulating of scientific and effective management strategies. In the face of challenges such as climate change and resource limitations, the promotion of this technology is of significant importance to the sustainable development of agriculture.

Further optimization remains a crucial research direction. Considering the diversity of crops and the complexity of growing environments, the adaptability, durability, and data collection capabilities of the sensor need to be improved, particularly in meeting the demands of different crops and growth stages. Additionally, integrating Internet of Things (IoT) and big data technologies will expand the application of wearable sensors in agricultural supply chains, promoting the comprehensive development of smart agriculture.

## 5 Conclusions

This study verifies the feasibility of the designed flexible spectral sensor for plant leaves, especially in its application for monitoring plant physiological characteristics. By integrating the light source with the AS7343 spectral sensor, an active multi-wavelength solution for near-ultraviolet, visible, and near-infrared light was achieved. This design not only improves the sensor’s flexibility but also enhances its adaptability across different spectral ranges. During the experimental process, the spectral sensor was installed on the underside of the leaf using a magnetic connection, minimizing the impact on leaf photosynthesis and respiration during long-term data collection. The original spectral reflection data of the leaves, collected through the AS7343 spectral sensor, was combined with information from the Clear band to compute the original relative spectral data. Various processing methods, including machine learning, denoising preprocessing, and multi-model fusion, were employed for the original spectral data and original relative spectral data to complete the regression prediction analyses of leaf water content and chlorophyll concentration. Notably, for the leaf chlorophyll in the original relative spectrum, the Gaussian Process Regression achieved the best prediction result during multivariate scatter correction, with a R_c_² of 0.8261 and RMSE_c_ of 1.7444 on the training set; on the test set, R_p_² was 0.7155, with RMSE_p_ of 2.0374. Meanwhile, for leaf water content, under various preprocessing conditions of the relative spectrum, Gradient Boosting Regression was able to make effective predictions, with R_c_² of 0.9401 and RMSE_c_ of 0.0028 on the training set; on the test set, R_p_² was 0.6667, with RMSE_p_ of 0.0067.

## Data Availability

The original contributions presented in the study are included in the article/**Supplementary Material**. Further inquiries can be directed to the corresponding author.
